# Prevalence of Depression in Medical Students at the Lebanese University and Exploring its Correlation With Facebook Relevance: A Questionnaire Study

**DOI:** 10.2196/resprot.4551

**Published:** 2016-05-31

**Authors:** Wadih J Naja, Alaa H Kansoun, Ramzi S Haddad

**Affiliations:** ^1^ Department of Psychiatry Faculty of Medical Sciences Lebanese University Beirut Lebanon; ^2^ Faculty of Medical Sciences Lebanese University Beirut Lebanon

**Keywords:** depression, Facebook, PHQ-9, FbRQ, Lebanese University Faculty of Medicine

## Abstract

**Background:**

The prevalence of major depression is particularly high in medical students, affecting around one-third of this population. Moreover, online social media, in particular Facebook, is becoming an intrinsic part in the life of a growing proportion of individuals worldwide.

**Objective:**

Our primary objective is to identify the prevalence of depression in medical students at the Lebanese University Faculty of Medicine, a unique state university in Lebanon, its correlation with the utilization of the interactive features of Facebook, and the way students may resort to these features.

**Methods:**

Students of the Lebanese University Faculty of Medicine were assessed for (1) depression and (2) Facebook activity. To screen for major depression, we used the Patient Health Questionnaire-9 (PHQ-9) scale. To test for Facebook activity, we developed the Facebook Resorting Questionnaire (FbRQ), which measures the degree to which students resort to Facebook.

**Results:**

A total of 365 out of 480 students (76.0%) participated in the survey. A total of 25 students were excluded, hence 340 students were included in the final analysis. Current depression was reported in 117 students out of 340 (34.4%) and *t* tests showed female predominance. Moreover, PHQ-9 score multiple regression analysis showed that feeling depressed is explained 63.5% of the time by specific independent variables studied from the PHQ-9 and the FbRQ. Depression varied significantly among the different academic years (*P*<.001) and it peaked in the third-year students. One-way analysis of variance (ANOVA) showed that depression and resorting to Facebook had a positive and significant relationship (*P*=.003) and the different FbRQ categories had significant differences in resorting-to-Facebook power. The like, add friend, and check-in features students used when resorting to Facebook were significantly associated with depression.

**Conclusions:**

This study showed that depression was highly prevalent among students of the Faculty of Medicine at the Lebanese University. Moreover, Facebook may be a promising, helpful, psychological tool for optimizing the management of depression. Our study brought to bear further questions that now prompt further observation and scrutiny to know more about the high rates of depression in this student population, more so in the part of the world studied, and to the growing role of social media.

## Introduction

### On Depression

Depression was shown to have high prevalence rates among university students [1,2]. These rates were even higher when it came to medical students, where depression affected one-third [3] to one-half of this population [4]. Coping strategies, such as social support, were recommended for medical students to improve their quality of life [5]. Moreover, it was shown that social support per se may even be a potent protective factor for depression [6].

### On Facebook

On average, there are about 864 million daily active users on Facebook, the most visited online social network (OSN) in the world [7]. Kross et al showed that Facebook negatively affected a person's well-being in two ways: affective well-being (ie, how he/she feels moment to moment) and cognitive well-being [8]. In addition, individuals who have used Facebook accounts longer and users who have a greater number of nonpersonal Facebook friends believe that others' lives are better and are even more pleasant than their own [9]; this and other Facebook-mediated comparisons were associated with greater depression [10]. Furthermore, Forest et al reported that people with low self-esteem will not take advantage of the easy means of disclosure on Facebook, despite their beliefs in such a possibility [11]. In addition, it was reported that the increased usage of Facebook photo applications is correlated with body image disturbance and weight dissatisfaction [12].

As a part of *infodemiology* [13], which is a new method to study health determinants and information on the Internet, Facebook is one possible screening method to study health-related issues. For instance, a previous study based on verbatim text analysis showed that displayers of depressive symptoms are more likely than nondisplayers to have a positive Patient Health Questionnaire-9 (PHQ-9) measure [14].

Furthermore, announcing suicide on Facebook could provide enough time for an intervention [15]. Also, Facebook may be a good means for individuals to disclose their depression, hence enabling others to constantly be updated [16]. Seemingly depressed participants in Social Network Service (SNS) had the opportunity to communicate with peers that they selected. Such behavior may have been promoted by anonymity, hence avoiding face-to-face contact [17].

Finally, depressed people had a declined usage of Facebook features such as *location tagging*, *add friend*, and the *like* feature [18]. However, we were concerned more with the increased usage of Facebook as a possible tool that students could resort to in fighting depression. From this perspective, a subjective report of how each feature is resorted to might be more significant than counting the usage of each. Hence, when it comes to depressed Facebook users, we questioned whether resorting to Facebook may be a means to relieve depressive symptoms.

### Objectives

Our primary objective was to study the prevalence of major depression in medical students attending the only state university in Lebanon and its correlation with these students resorting to Facebook.

## Methods

### Participants

Participants were medical students from the second up to the fifth year who attended the Faculty of Medicine at the Lebanese University. Sixth- and seventh-year students could not be included since they were in clinical rotations in several hospitals. Taking into consideration that all students at the Faculty of Medicine had taken an advanced English course and since lectures are delivered in English, we distributed the questionnaires in English. The survey extended from February 6-25, 2014. We visited each class twice on two consecutive days in order to guarantee maximum attendance.

### Questionnaires

#### Patient Health Questionnaire-9

The PHQ-9 is a reliable and valid criteria-based diagnostic tool for depressive disorders and is used to measure the severity of depression [19]. Indeed, this scale has a high specificity (94%) and a relatively low sensitivity (73%) [19]. Up to the date of our field work, the PHQ-9 was not validated in the Lebanese population.

#### Facebook Resorting Questionnaire

Previous reports focused on one of two things in screening Facebook features related to depression: (1) declined Facebook activity [18] or (2) disclosures on Facebook that showed depression [14-16]. We assembled a new questionnaire—the Facebook Resorting Questionnaire (FbRQ)—that aimed to study the tendency of users to resort to Facebook features and the objectives of their usage. The FbRQ assesses the reasons users eventually resort to Facebook features during a current depressive state. Our questionnaire was not designed to measure time spent on Facebook or how many times each feature is used.

The FbRQ was comprised of seven questions targeting the different features of Facebook and the purpose underlying their utilization:

1. Question about the *like* feature: “Do you like topics on Facebook that you couldn’t own, meet, visit...in your real life?” What mattered was whether this feature allowed the user to stay up to date with the subject of interest.

2. Question about the *location-tagging* feature: “Do you have the habit of tagging your current location wherever you go?” This tag feature used by Facebook friends could provide the user with the possibility to navigate new locations and to check them.

3. Question about the *disclosure* feature: “Do you feel that Facebook is a way to express your feelings or whatever you want to declare in your real life and you do better in expressing on Facebook than on a real scale?” The disclosure feature might provide the user with a basis for social support and electronic feedback that could become a source for generating new ideas.

4. Question about the *add friend* feature: “When you want to meet someone, do you feel that Facebook, by the feature *add friend* and further chatting, makes you more comfortable than a verbal meeting?” This question and answer provided an understanding about the clinical importance of depression and whether the user found it easier to add and meet friends on Facebook compared to real life.

5. Question about the *photo* feature: “Do you think that Facebook, by posting photos, saves your best life moments and helps you evoke your positive memories?” Posting photos is another feature that users tend to use. Sticking to our objectives, we aimed to detect whether this feature helped the user recall positive memories that the user would then ruminate about.

6. Question about the aim behind using Facebook: “Is Facebook a way to show the best out of your life?”

7. Question about the aim behind using Facebook: “Does Facebook render your voice audible and your existence noticeable?” Questions 6 and 7 aimed to help us understand the motives behind the use of Facebook.

The FbRQ was chiefly designed to cover all the above-mentioned points of every Facebook feature and aim of usage. Multimedia Appendix 1 is a snapshot that shows the FbRQ. Each question was given 1 point if answered positively, for a total of 7 points. We decided to divide users resorting to Facebook into three categories: low (0-1 points), moderate (2-3 points), or high (4-7 points). We also collected some sociodemographic data concerning the following: gender, academic year, psychiatric history, and whether users were smokers or nonsmokers, among other criteria.

### Survey Leaflet

The survey was totally anonymous. The ethics committee at our university required that we provide clear information to the participants prior to their written consent (see Multimedia Appendices 2 and ).

### Statistical Analysis

Statistical analysis was conducted using IBM SPSS Statistics for Windows version 16.0 (IBM Corp). Correlation analysis between depression and resorting to Facebook, along with sociodemographics, was performed using the chi-square test for which we calculated the *P* value and Cramer's V value, which is the appropriate measure of association. We performed *t* tests to show the predisposition to depression concerning gender. Furthermore, one-way analysis of variance (ANOVA) was conducted to determine the significance of the relationship between resorting to Facebook and depression, the variability of depression among the different academic years, as well as determining a significant difference between each FbRQ category. Moreover, multiple regression analysis was conducted to examine the relationship between different variables in the study for which we calculated the R^2^value, which reflects the degree of correlation, and the *F* statistic, which tells us if the model is a good fit. Statistical significance for hypothesis testing was set at 5% (*P*<.05).

## Results

### Overview

A total of 365 of 480 students (76.0%) took the survey; the remaining students were absent on the days of our survey. A total of 25 students out of 365 (6.8%) were excluded from the study because some did not complete the three questionnaires; 9 of these students refused to participate. Hence, we included a total of 340 students in the study. The sociodemographic characteristics of the sample are summarized in Table 1.

**Table 1:** Sociodemographic characteristics of the sample (n=340).

Characteristics	n (%)
Participants who took the survey (n=480)	365 (76.0)
Participants with depression	117 (34.4)
Males	145 (42.6)
Females	195 (57.4)
Participants with functional impairment	256 (75.3)
Participants living in dorms	196 (57.6)
Married	3 (0.9)
Smokers	35 (10.3)
Participants with psychiatric history	62 (18.2)

**Figure 1:**
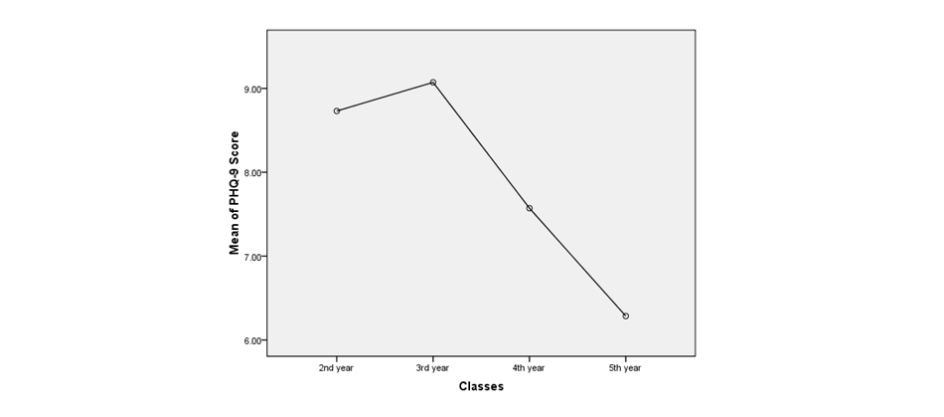
Mean depression score by class year. PHQ-9: Patient Health Questionnaire-9.

On the PHQ-9 scale, for a cut-off of 10, the prevalence rate of depression was 34.4% (117/340). Severity was distributed as follows: out of 117 students, 5 (4.3%) had severe depression, 32 (27.4%) had moderate depression, and 80 (68.4%) had mild depression. Suicidal thoughts in depressed students—represented by Question 9 on the PHQ-9 scale—were attributed to 27.4% (32/117) of students. Table 2 shows the multiple regression analysis conducted between Question 2 of the PHQ-9 as a dependent variable and Questions 1, 3, 4, 6, 8, 9, and 10 of the PHQ-9 and Question 2 of the FbRQ as independent variables. The R^2^value reached 63.5%, the *F* statistic showed that the model was a good fit, and this regression was a good model (*P*<.001). As plotted in Figure 1 and as seen in Table 3 , after a one-way ANOVA, depression scores averaged 8.7, 9.0, 7.5, and 6.2, and depression reached 41.5%, 46.9%, 31.6%, and 21.4% in the second through the fifth year, respectively. The calculated value of *F* was 6.1 (*P*<.001). Comparing the different academic year classes, the statistical significance was owed only to the relationship between year 5 with respect to second- and third-year students (*P*<.05).

**Table 2:** Multiple regression analysis and the significance of the independent variables in affecting the dependent variable.

Model	Unstandardized coefficients		Standardized coefficients	*t*	*P*
	B	SD	Beta		
(Constant)	.051	.081	N/A^a^	0.630	.52
FbRQ^b^Question 2	.314	.149	.091	2.109	*.03* ^c^
PHQ-9^d^Question 1	.192	.043	.210	4.456	*<.001*
PHQ-9 Question 3	.062	.038	.077	1.616	.10
PHQ-9 Question 4	.155	.044	.168	3.532	*<.001*
PHQ-9 Question 6	.184	.046	.197	3.988	*<.001*
PHQ-9 Question 8	.073	.051	.066	1.440	.10
PHQ-9 Question 9	.108	.059	.083	1.845	.06
PHQ-9 Question 10	.281	.062	.209	4.518	*<.001*

^a^N/A: not applicable.

^b^FbRQ: Facebook Resorting Questionnaire.

^c^Significant values are shown in italics (*P*<.05).

^d^PHQ-9: Patient Health Questionnaire-9.

**Table 3:** Variation of depression means with respect to the different classes, and model analyses (n=340).

Classes or models		Number of students, n (%)	Depression mean	SD	SE	95% CI
**Class**						
	Second year	89 (26.2)	8.7303	4.1609	0.4411	7.8538-9.6068
	Third year	83 (34.4)	9.0723	4.8459	0.5319	8.0142-10.1304
	Fourth year	98 (28.8)	7.5714	4.6061	0.4653	6.6480-8.4949
	Fifth year	70 (20.6)	6.2857	4.2054	0.5026	5.2830-7.2885
	Total	340 (100)	7.9765	4.5745	0.2481	7.4885-8.4645
**Model**						
	Fixed effects	N/A^a^	N/A	4.4746	0.2427	7.4991-8.4538
	Random effects	N/A	N/A	N/A	0.6042	6.0538-9.8992

^a^N/A: not applicable.

### Patient Health Questionnaire-9 and Sociodemographic Parameters

Among sociodemographics, the chi-square test showed a significant relationship between depression and gender only (*P*=.007), as shown in Table 4. In addition, Cramer's V value was significant (*P*=.007) and equaled .189. To determine which gender was more predisposed to depression, a *t* test was performed and showed that females had higher rates of depression than males with a factor of 1.8, as shown in Table 5.

**Table 4:** Depression categories distributed by gender (n=340).

Participants		Depression category^a^, n (%)				Total, n (%)	Pearson chi-square *P* value
		<10	10-14	15-19	≥20		
**Gender**							
	Male	109 (32.1)	28 (8.2)	7 (2.1)	1 (0.3)	145 (42.6)	.007^b^
	Female	114 (33.5)	52 (15.3)	25 (7.4)	4 (1.2)	195 (57.4)	N/A^c^
Total		223 (65.6)	80 (23.5)	32 (9.4)	5 (4.5)	340 (100)	N/A

^a^Level of depression divided into the following categories, with scores in parentheses: *no depression* (<10), *mild* (10-14), *moderate* (15-19), and *severe* (≥20).

^b^Significant values are shown in italics (*P*<.05).

^c^N/A: not applicable.

**Table 5:** Difference in depression between males and females, and *t* tests (n=340).

Participants		n (%)	Depression mean	SD	SE
**Gender**					
	Male	145 (42.6)	6.9310	4.2617	0.3539
	Female	195 (57.4)	8.7538	4.6541	0.3333

### Patient Health Questionnaire-9 and Facebook Resorting Questionnaire

Table 6 is a result of the one-way ANOVA showing that, out of 340 students, resorting to Facebook was high in 61 students (17.9%), moderate in 149 students (43.8%), and low in 130 students (38.2%). It was shown that there is a positive and significant relationship between the depression mean and the categories related to resorting to Facebook, whereby the depression mean was 6.9 in the low FbRQ group, 8.4 in the moderate group, and 9.0 in the high group. The *F* value was 5.843 (*P*=.003). Comparing the different FbRQ groups, the relationship was significant between the low and moderate groups (*P*=.03), and between the low and high groups (*P*=.01); however, it was not significant between the moderate and high groups.

**Table 6:** Variation of resorting to Facebook with respect to depression mean (n=340).

Groups or models		n (%)	Depression mean	SD	SE	95% CI
**FbRQ**^a^ **groups**						
	Low	130 (38.2)	6.9615	4.3122	0.3782	6.2132-7.7098
	Moderate	149 (43.8)	8.4027	4.5291	0.3710	7.6695-9.1359
	High	61 (17.9)	9.0984	4.8673	0.6232	7.8518-10.3449
	Total	340 (100)	7.9765	4.5745	0.2481	7.4885-8.4645
**Model**						
	Fixed effects	N/A^b^	N/A	4.5105	0.2446	7.4953-8.4576
	Random effects	N/A	N/A	N/A	0.6332	5.2523-10.7007

^a^FbRQ: Facebook Resorting Questionnaire.

^b^N/A: not applicable.

There was no statistically significant relationship between the sociodemographic parameters studied and the FbRQ results as determined by the chi-square test. However, a regression analysis was performed locking the PHQ-9 score as a dependent variable and all the Facebook features as the independent ones. The R^2^equaled 25.3%, the model was a good fit, and the regression was a good model (*P*<.001). Table 7 shows the different ratios and the significance of each independent variable.

**Table 7:** Different ratios and significance of each independent variable.

Model	Unstandardized coefficients		Standardized coefficients	*t*	*P*
	B	SD	Beta		
(Constant)	.893	.065	N/A^a^	13.83	*<.001* ^b^
FbRQ^c^Question 1	.153	.072	.093	2.13	*.03*
FbRQ Question 2	.617	.148	.181	4.17	*<.001*
FbRQ Question 3	-.102	.100	-.046	-1.02	.30
FbRQ Question 4	.191	.086	.095	2.23	*.02*
FbRQ Question 5	-.085	.076	-.051	-1.11	.26
PHQ-9^d^Question 6	.150	.093	.073	1.61	.10
PHQ-9 Question 7	.131	.087	.071	1.50	.13

^a^N/A: not applicable.

^b^Significant values are shown in italics (*P*<.05).

^c^FbRQ: Facebook Resorting Questionnaire.

^d^PHQ-9: Patient Health Questionnaire-9.

## Discussion

### On Depression

The current prevalence of depression in our sample (34.4%) is slightly higher than what is reported in the literature, which is around 30% [1,2]. Studies on Question 9 of the PHQ-9 showed the suicidal ideation in the studied sample to be lower than in previous reports (23%) [20]. Nevertheless, it is always considered high since a significant proportion of people reporting suicidal ideation accounted for half of fatal or nonfatal suicide attempts [20]. Multiple regression analyses showed the question of depressive mood (Question 2), locked as the dependent variable, to correlate highly with other PHQ-9 items; hence, reports of feeling down or depressed by the students was explained 63.5% of the time by all the independent variables studied. Among these variables, the problems of interest in doing things, fatigue, regarding one's self as a failure, and functionality were positively and significantly correlated and, thus, each 1% increase in these problems will increase feelings of depression by a factor of 0.19%, 0.15%, 0.1%, and 0.28%, respectively. On the other hand, a 1% increase in resorting to the *check-in* Facebook feature was associated with a 0.31% increase in feeling depressed, as shown in Table 2. Moreover, depression varied significantly among the different academic year classes and it peaked in the third year, which can be explained by the female predominance, then decreased gradually to a minimum PHQ-9 average of 6.2 in the fifth year. Female predominance was observed with a factor of 1.8, which is consistent with other reports [6,14,21]. From Cramer's V value of .189, we can conclude that this significant relationship was low to moderate. Finally, our results regarding family history as a predisposing factor is not consistent with the literature [6].

### On Resorting to Facebook

#### Overview

About one-third of students with Facebook accounts are active on Facebook [14,16]. Moreover, depressed users are expected to exhibit a decreased usage of Facebook [18]; however, in our sample a depressed user had an increased need to resort to Facebook. The one-way ANOVA came to show that as the mean of depression increases, resorting to Facebook increases as well (*P*=.003). Hence, depressed medical students at the Faculty of Medicine are more likely than the nondepressed students to resort to Facebook, which leads to the hypothesis that Facebook may be a coping mechanism for depression. Moreover, this analysis provides credibility for the FbRQ by showing that the difference in resorting to Facebook is significant between each and every FbRQ category, except when it comes to comparing the moderate and high categories. Unlike prior studies [14] where resorting to Facebook was higher among females, there was no predilection to resorting to Facebook among genders.

What is common to the features surveyed by the FbRQ scale is that resorting to Facebook provides some pattern of social life without necessarily improving deficient communication skills. Users' subjective reports on the FbRQ show that Facebook may provide users who have a tendency to experience depression a chance to stay in contact with topics of interest, new places, and horizons; to ruminate on positive memories; express ideas; meet friends; and show the best out of their lives. According to the multiple regression analysis, resorting to the *like*, *add friend*, and *check-in* features was associated with depression; for each 1% increase in their usage, the PHQ-9 scores increased by 0.1, 0.6, and 0.1%, respectively. Hence, Facebook could have a positive psychosocial impact by allowing users to resort to these features.

#### Facebook in Relation to Psychotherapy

Interpersonal therapy aims to (1) improve communication skills and (2) increase self-esteem [22]. Facebook improved depression in university students [23] by expressing the opinions of a depressed person that are usually suppressed on a daily basis; this was demonstrated by Questions 6 and 7 on the FbRQ, in addition to the *add friend* feature, chatting, and the *like* feature.

On the other hand, Facebook was shown to clearly improve self-esteem [24]. Indeed, Internet use improved self-esteem as well as social support, while decreasing the percentage of depression and loneliness [25]. This seems to be linked to the *add friend* feature and the aim of the usage of Facebook as per the responses to the FbRQ.

Facebook, as a humanistic therapy, provides a social environment where individuals may compare old and new habits or attitudes with friends. At the same time, individuals may find out that similar problems may also be encountered by others, hence providing some kind of relief [26]. In conclusion, psychotherapies are providing hope, empathy, and care; hence, individuals who are supported by close friends and caring people are less likely to need or seek therapy [26].

#### Facebook Social Support

Notably, social support is a key protective factor against depression [6]. Computer-mediated interaction has been shown to improve a user's confidence over the course of a conversation and strengthens judgments while negotiating. Most importantly, it helps a person get rid of uncertain behaviors [27]. On the other hand, lonely people find it hard to introduce themselves to, or take the first step in meeting, new people [4]. Facebook may be an option to overcome those difficulties by allowing an individual to choose the contact of his/her preference.

### Study Limitations

As a cross-sectional study, our results were confined to comparing, and not eliciting, a cause-and-effect relationship between depression improvement and resorting to Facebook. The standardized PHQ-9 questionnaire had not been validated in the Lebanese population during our survey. On the other hand, the FbRQ is a novel tool that we developed due to the lack of an equivalent standardized questionnaire; its point of weakness lies in not using a continuous variable scale.

### Conclusions

Depression was shown to be highly prevalent among medical students of the Lebanese University. Moreover, depressed Facebook users were more likely than nondepressed users to resort to Facebook; the subjective report by the FbRQ allows us to hypothesize that Facebook may be a means to alleviate depressive symptoms. Specifically, among all the Facebook features, *like*, *check-in*, and *add friend* may be of therapeutic significance. These results should stimulate health care providers to question the high depression rate in this student population and to acknowledge the possible role of Facebook in a multidisciplinary treatment strategy to relieve depression.

## Appendix

Multimedia Appendix 1 Snapshot of the Facebook Resorting Questionnaire (FbRQ).

Multimedia Appendix 2 Survey cover showing the contents.

Multimedia Appendix 3 Survey introduction.

## Abbreviations

ANOVA: analysis of variance
FbRQ: Facebook Resorting Questionnaire
N/A: not applicable
OSN: online social network
PHQ-9: Patient Health Questionnaire-9
SNS: Social Network Service
