# Supplementary material for: Prevalence of Depression in Medical Students at the Lebanese University and Exploring its Correlation With Facebook Relevance: A Questionnaire Study
Source: JMIR Res Protoc. 2016 May 31;5(2):e96. doi: 10.2196/resprot.4551 (PMC4908302; doi:10.2196/resprot.4551)
Supplement: Multimedia Appendix 1 [file resprot_v5i2e96_app1.pdf]

Facebook Resorting Questionnaire (FbRQ)

Do you have a facebook account?	**No**	**Yes**

Mark your answer by a signal.

	**No**	**Yes**
**1.** Do you like topics on fb that you couldn't own, meet, visit.. in your real life?	0	1
**2.** Do you have the habit to tag your current location wherever you go?	0	1
**3.** Do you feel that fb is a way to express your feelings or whatever you want to declare in your real life and you do better in expressing on fb than on real scale?	0	1
**4.** When you want to meet someone, do you feel that fb, by the feature 'add friend' and further chatting, make you more comfortable than verbal meeting?	0	1
**5.** Do you think that fb- by posting photos- save your life-best-moments and help you evoke your positive memories?	0	1
**6.** Is fb a way to show the best out of your life?	0	1
**7.** Does fb render your voice audible and your existence noticeable?	0	1

TOTAL:
