# Supplementary material for: Prevalence of Depression in Medical Students at the Lebanese University and Exploring its Correlation With Facebook Relevance: A Questionnaire Study
Source: JMIR Res Protoc. 2016 May 31;5(2):e96. doi: 10.2196/resprot.4551 (PMC4908302; doi:10.2196/resprot.4551)
Supplement: Multimedia Appendix 2 [file resprot_v5i2e96_app2.pdf]

File Number:

Facebook and Depression

Paper contents:

I> Consent

II> PHQ-9 scale

III> FBAQ scale

IV> SD scale
