# Supplementary material for: Prevalence of Depression in Medical Students at the Lebanese University and Exploring its Correlation With Facebook Relevance: A Questionnaire Study
Source: JMIR Res Protoc. 2016 May 31;5(2):e96. doi: 10.2196/resprot.4551 (PMC4908302; doi:10.2196/resprot.4551)
Supplement: Multimedia Appendix 3 [file resprot_v5i2e96_app3.pdf]

The goal of this study is to detect a possible preventive role of facebook against depression. For this, we provided you this booklet that is composed to 3 questionnaires:

- ✓ PHQ-9 scale that measures the **tendency** to develop depression, **if any**;
- ✓ FBAQ scale which measures the facebook activity.
- ✓ SDQ scale

Note that each participant is **anonymous** and not any clue, from the booklet, will tell about the identity.

Hence feel free to fill these 3 scales; it won't take you much time but it may mean a lot on larger scientific scales. It is your right whether to participate or not and your refusal will not harm you in anyway. Your participation will add value on top of the scientific meaning. You can terminate at any point.

Kindly mark a signal if you agree to take it.

◯ *I Agree*

◯ *I Disagree*

In case you disagree, please inform us about the reason(s).

____

____

____
